# One Bloom Is Not Like the Other—Distinct Environmental Drivers Result in Domoic Acid Events in Monterey Bay, California

**DOI:** 10.3390/toxins17100511

**Published:** 2025-10-17

**Authors:** Aubrey Trapp, Andrew Baker, Kendra Hayashi, Raphael M. Kudela

**Affiliations:** 1 Department of Ocean Sciences, University of California, 1156 High Street, Santa Cruz, CA 95064, USA; khayashi@ucsc.edu (K.H.); kudela@ucsc.edu (R.M.K.); 2 Waters Corporation, 5720 Stoneridge Dr., Pleasonton, CA 94588, USA; andy_baker@waters.com

**Keywords:** domoic acid, harmful algal blooms, solid-phase adsorption toxin tracking, SPATT, environmental metabolomics, HAB monitoring

## Abstract

Domoic acid (DA), produced by *Pseudo-nitzschia* diatoms, is the one of the major toxin threats from harmful algal blooms (HABs) on the west coast of the United States. DA events vary in magnitude, timing, and duration, and elucidating drivers for individual events is a persistent challenge. Monterey Bay experiences near-annual DA events and hosts long-term HAB monitoring at the Santa Cruz Municipal Wharf (SCW). Here we characterize two toxin events, occurring in May 2023 and March 2024. The events were similar in magnitude and duration, but an exploration of physical, biological, and chemical dynamics revealed distinct environmental drivers. These differences resulted in a significant deviation in cellular DA (cDA) within the same species of *Pseudo-nitzschia*. In addition, opportunistic solid-phase adsorption toxin tracking (SPATT) was used for environmental metabolomics. The novel application of SPATT revealed 159 metabolites that were strongly correlated with DA in both events and produced a spectral match to a new marine natural product using Global Natural Products Social Molecular Networking (GNPS). This work takes a multivariable approach to understanding toxin drivers and lends proof of concept for the integration of environmental metabolomics in HAB monitoring.

## 1. Introduction

Harmful algal blooms (HABs) occur when phytoplankton growth causes negative impacts on marine and freshwater systems. HAB mechanisms may be species specific, such as the production of toxins, or general, such as damage to fish gills and deoxygenation in shallow waters [1]. On the west coast of the United States, marine HABs are commonly caused by *Pseudo-nitzschia* diatoms [2]. Toxigenic species produce domoic acid (DA), a glutamate receptor agonist and potent neurotoxin [3]. About half of the 60 currently reported *Pseudo-nitzschia* species are known to produce DA with a high possibility of additional discoveries [4]. As with other algal biotoxins, DA is transferred through marine food webs via bioaccumulation in lower trophic levels. Vectors include shellfish, crustaceans, squid, and bait fish, among others [5,6,7]. DA poisoning, called amnesiac shellfish poisoning (ASP) in humans, has resulted in mass mortality of sealions and marine birds [8,9] and cardiac disease in Southern sea otters on the California coast [10]. DA was also detected in 13 Alaskan marine mammal species, though the extent of negative health effects at low concentrations is largely unknown [11]. International safety guidelines prohibit the distribution of seafood containing more than 20 ppm of DA. However, seafood consumers, especially recreational anglers and shellfishers, face chronic low-dose exposure [3,12]. DA is also a socio-economic threat to coastal communities. In 2008, the cost of a full-season razor clam closure in Washington was estimated at $20.4 million [13]. Additionally, an extreme *Pseudo-nitzschia* HAB in 2015 cost the Dungeness crab fishery nearly $100 million, and tourism losses amounted to $40 million (NOAA fisheries) [14]. These threats make clear the urgent need for holistic understanding of HAB drivers on the United States west coast.

Marine HABs are notoriously difficult to predict because of the vast diversity of factors influencing phytoplankton dynamics. Efforts to elucidate drivers of *Pseudo-nitzschia* HABs in the California Current System (CCS) have identified strong associations to nutrient limitation and climate variability, though there is still uncertainty in direct links between environmental conditions and DA [15]. As an eastern boundary current, the prevailing seasonality in the CCS is forced by alongshore winds. Southward winds initiate the upwelling season during spring and summer, driving surface water offshore and supplying deep nutrients to the coast [16,17]. During fall and winter, wind relaxations and reversals become more frequent, and upwelling ceases. Relatively warm, offshore water flows along the surface toward the coast [17]. Large-scale climate variability from El Niño Southern Oscillation (ENSO) reduces the magnitude of upwelling, which can affect the timing and strength of the spring transition. El Niño works to sever surface waters from deep nutrient sources during spring, however elevated rainfall and storm activity may increase nutrient supply from coastal runoff and sediment resuspension [18].

Linking *Pseudo-nitzschia* HABs to the physical environment is further complicated because different regions exhibit contrasting responses to the same drivers [19,20]. In addition, inter- and intra-specific variation in toxin production adds uncertainty to predicting the magnitude of a toxin event. At least 9 toxigenic *Pseudo-nitzschia* species have distributions in the CCS with multiple species often contributing to particulate DA (pDA) concentrations [21,22]. DA production is also regulated by allelopathic interactions with other organisms. For example, toxin production has been found to increase in response to chemical cues from copepod grazers [23,24], and DA appears to interplay with the bacterial community during bloom succession [25]. Taken together, physical, biological, and chemical factors in the environment create a combinatorial challenge for elucidating DA drivers in the CCS.

The present study aims to characterize distinct DA events through an investigation of multiple *Pseudo-nitzschia* HAB drivers. We focus on toxin events recorded at the Santa Cruz Municipal Wharf (SCW) in 2023 and 2024. A key feature of this site is the Monterey Bay upwelling shadow, which creates a retentive circulation pattern in the northwest corner of the bay during upwelling [26]. Under relaxation dynamics, the upwelling shadow breaks down, flushing the region with offshore, surface water [26]. This feature makes the SCW a useful “field lab” for studying bloom dynamics across different physical regimes [27,28]. The SCW is also advantageous because it hosts a long term timeseries of co-located weekly measurements of pDA and phytoplankton abundance along with a suite of physical data. To profile the chemical environment during DA events, we apply a novel untargeted approach to opportunistically sample environmental metabolomes using solid-phase adsorption toxin tracking (SPATT). SPATT is a resin-based, passive sampling device commonly used to measure dissolved HAB toxins [29]. Diaion^®^ HP20 resin is high capacity and widely adsorbent over a range of lipophilicity, which supports tracking multiple metabolites simultaneously [30]. The integration of environmental metabolomics, which analyzes all detectable metabolites in a sample [31], with HAB monitoring has potential to provide a wealth of new data on bloom dynamics and toxin production.

## 2. Results

### 2.1. Toxin Events

**Figure 1:**
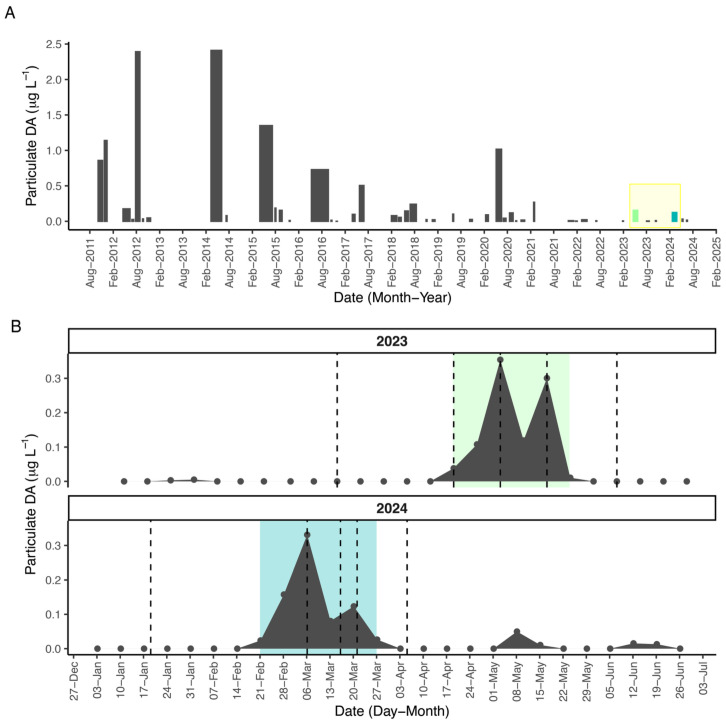
(**A**) Magnitude and duration of SCW toxin events from 2011–2024. (**B**) Weekly pDA for the largest events in 2023 and 2024, respectively. Dotted lines indicate SPATT samples for untargeted metabolomics. Green shading indicates the toxin event period in 2023 and blue shading indicates the toxin event period in 2024.

Beginning in 2011, long-term monitoring at the SCW revealed near annual DA events in Monterey Bay (Figure 1A). The largest events in 2023 and 2024 were similar in magnitude and duration (Table 1). Historically, these are relatively small increases in DA, reaching only 53% (2023) and 42% (2024) of the mean of all events from 2011–2024, though the duration was about 1.5 weeks longer than average (Table S1). In 2023, toxin peaked at 0.35 µg L^−1^ pDA on May 3, and the 2024 event peaked at 0.33 µg L^−1^ on 6 March 2024 (Figure 1B). The events differed in timing, with the 2024 event occuring nearly two months earlier than 2023. Small toxin increases were also detected in May of 2024, and later during summer for both years (Figure 1).

### 2.2. Physical Environment

**Figure 2:**
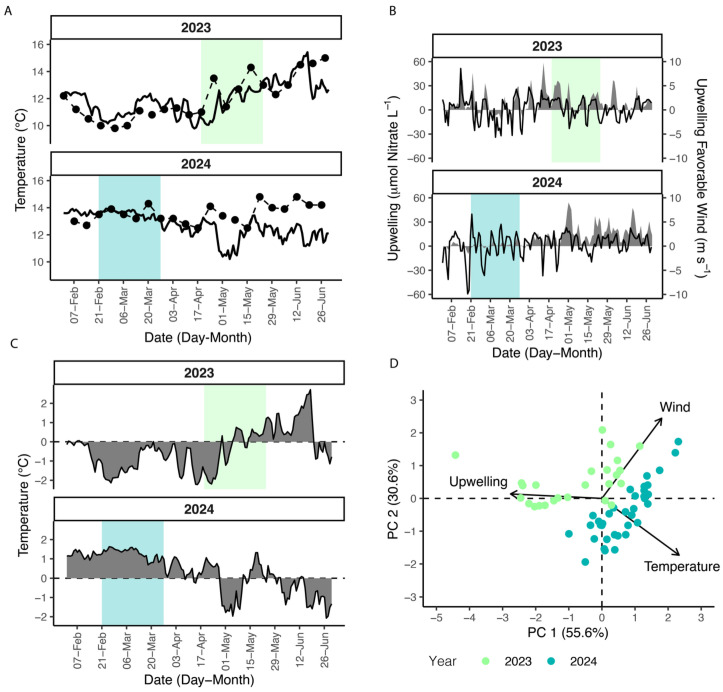
(**A**) Temperature from SCW (dashed) and NDBC 46092 (solid). (**B**) Biologically effective upwelling index (BEUTI) (gray ribbon) for 37° N and upwelling favorable winds at NDBC 46092 (black line). (**C**) Mean temperature anomaly at NDBC 46092. (**D**) Principal component biplot for data during DA event period. Green shading indicates the toxin event period in 2023 and blue shading indicates the toxin event period in 2024.

Toxin increases during both events coincided with warming temperature (Figure 2A). In the first week of pDA detection, temperatures at the SCW increased from 11.0–13.5 °C in 2023 and 13.5–13.9 °C in 2024. SCW temperatures tracked with offshore National Data Buoy Center (NDBC) 46092 in both years until mid-April, when measured temperature at the SCW rose 1–2 °C degrees relative to the buoy. Consistent with a global heat trend, the mean water temperature from February to June, 2024 was 0.7 °C warmer than in 2023 (Figure 2C). The mean temperature anomaly during the 2024 event was 1.28 °C, while the 2023 event anomaly was −0.33 °C cooler than climatology (Figure 2C).

As an eastern boundary current embayment, Monterey Bay is strongly influenced by wind-driven transport along the California coastline. Biologically effective upwelling transport index (BEUTI) and upwelling favorable winds trended together, and both toxin events initiated when alongshore winds were directed towards the south (Figure 2B). The 2023 toxin event started during a marked increase in upwelling. BEUTI showed nitrate flux increasing from about 5 µmol L^−1^ on April 19 to 31 µmol L^−1^ 2 days later. In contrast, the upwelling season of 2024 experienced a gradual ramp-up of upwelling with large pulses beginning in May. The 2024 toxin event occurred during a shift from net negative −9.570 µmol L^−1^ nitrate flux on Feb 19 to 2.255 µmol L^−1^ 2 days after the onset of the toxin event. Downwelling favorable winds occurred prior to the event, accounting for the negative nitrate flux. Increasing pDA coincided with a shift to weak upwelling favorable conditions, though pulses of downwelling winds continued through the 2024 event. Both events concluded as winds shifted again from south to north and upwelling declined. However, there was still net positive nitrate flux when pDA subsided.

Principal component analysis (PCA) of temperature, alongshore winds, and BEUTI during the toxin events explained 86.2% of the variance on two dimensions (Figure 2D). Years were differentiated on PC1 and PC2 with a majority of 2023 dates clustering towards negative PC1 and positive PC2. Dates in 2024 cluster in positive PC1 and negative PC2. Upwelling was a strong contributor to the negative mode of PC1, while alongshore winds and temperature are similarly correlated to PC1 and PC2.

### 2.3. Biological Environment

**Figure 3:**
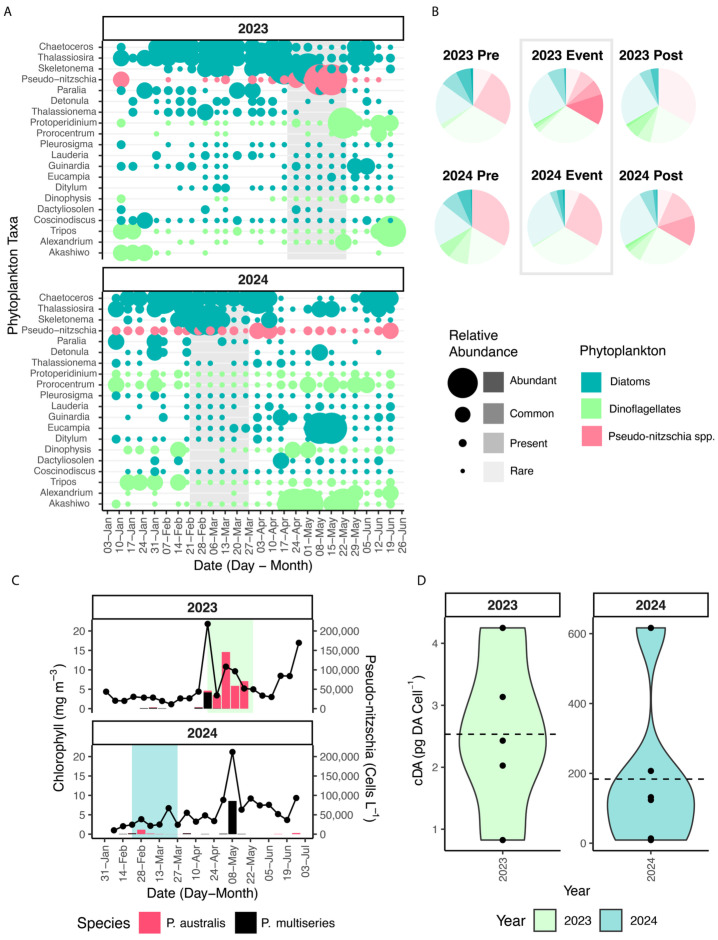
(**A**) RAI of the 20 most abundant taxa recorded at SCW. Point size indicates the RAI category, and genera are grouped into functional groups by color. RAI categories are: Abundant (≥ 50%), Common (10–49%), Present (1–9%), Rare (<1%). (**B**) Relative proportion of each functional group during the 35-day period before the event, during the event, and for 35 days after the event. Transparency indicates the RAI category. (**C**) Chl (black line) and *Pseudo-nitzschia* spp. (filled column) measured at SCW. (**D**) cDA calculated for each sampling date within each toxin event. The dashed line indicates the mean.

Phytoplankton biomass at the SCW was dominated by a mix of diatoms, including non-toxic genera *Chaetocerous* spp., *Thalassiosira* spp., and *Skeletonema* spp. as well as *Pseudo-nitzschia* spp. (Figure 3A). Succession to dinoflagellates, including high relative abundance of *Tripos* spp., *Akashiwo* spp., *Prorocentrum* spp., and *Protoperidinium* spp. were also observed during the study period (Figure 3A). Both years experienced a spring bloom in late April evidenced by a peak of *in situ* chlorophyll (Chl, Figure 3A). The 2023 toxin event coincided with an increase in *Pseudo-nitzschia* and Chl, indicating that toxigenic *Pseudo-nitzschia* was the dominant spring bloom taxa (Figure 3A,C). Interestingly, maximum *Pseudo-nitzschia* concentrations occurred during the April spring bloom in both years, but maximum pDA was measured prior to the Chl increase in 2024 (Figure 3C). Species probes showed that *P. australis* succeeded *P. multiseries* during the 2023 event, and the highest pDA concentrations coincided with the largest abundance of *P. australis* (Figure 3C). Cell counts reached nearly 150,000 cells L^−1^ during the height of the 2023 toxin event. Mean cellular DA (cDA) was estimated at 2.5 pg DA cell^−1^ and *Pseudo-nitzschia* standardized to Chl was 7560 cells µg Chl^−1^ (Figure 3C). A mixed community of diatoms preceded the 2023 event, and the *Pseudo-nitzschia* bloom gave way to *Protoperidinium* spp. and *Alexandrium* spp. dinoflagellates (Figure 3A,B). This contrasts with the 2024 toxin event, which was preceded by a mixed community of diatoms and dinoflagellates (Figure 3B). In addition, there was only a small increase in Chl and *Pseudo-nitzschia* did not exceed the present category in the relative abundance index (RAI) for the entire period of pDA detection. Rather, *Skeletonema* spp. and *Chaetocerous* spp. dominated the event, while dinoflagellates were more rare (Figure 3A). *P. australis* was also the causative species in this event, though abundance was low (Figure 3C). Cell counts during the event only reached 11,600 cells L^−1^, which drove up estimated cDA by two orders of magnitude relative to 2023 (183.6 pg DA Cell^−1^, Figure 3A,D). Overall biomass was lower, which resulted in a Chl standardization (866.8 cells µg Chl^−1^) that was approximately 1 order of magnitude lower than 2023 (Figure 3C,D). In the two weeks after pDA declined, *Pseudo-nitzschia* rose to common in the RAI (Figure 3B).

The spring bloom period for 2024 was also distinct from 2023, consisting of competing groups of diatoms, especially *Eucampia* spp., and dinoflagellates *Akashiwo* spp. and *Prorocentrum* spp. (Figure 3A). In contrast to the toxin events, *P. multiseries* was the dominant species during the 2024 spring bloom (Figure 3C). Using data from the tile installation at SCW, there was no statistical difference in color between the years (Chi-squared, *p* > 0.05), nor was there a discernable difference between event and non-event weeks (Figure S1).

### 2.4. Chemical Environment

**Figure 4:**
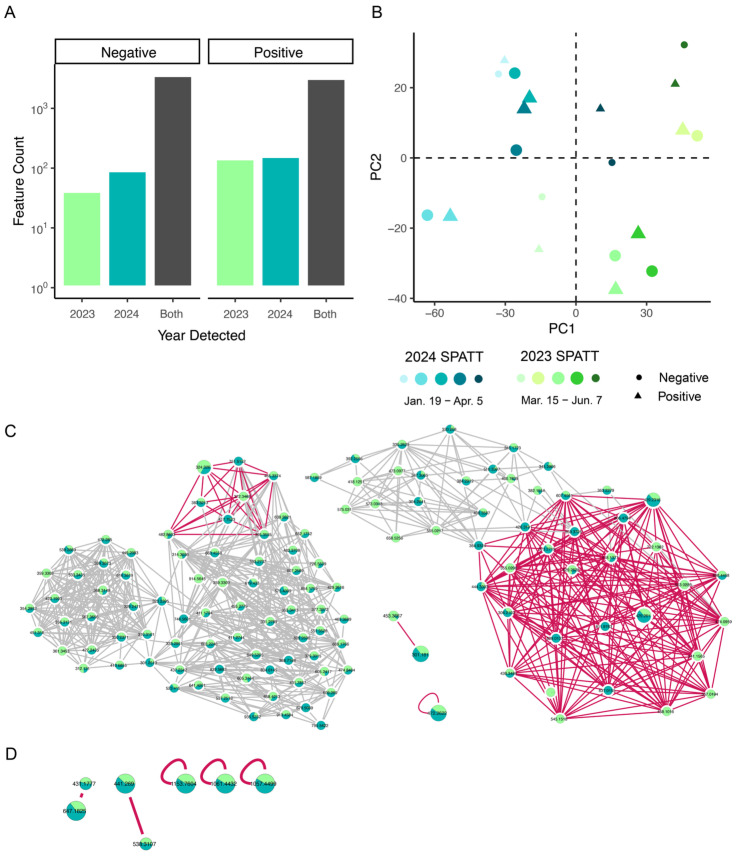
(**A**) Features identified by untargeted LCMS/MS. (**B**) PCA of feature intensity across two toxin events. Simplified molecular families containing the top 5 (**C**) positive mode and (**D**) negative mode features contributing to variance on PC1. Contributing features are shown larger and nearest neighbor edges are highlighted in pink. Pie charts show mean intensity for each feature in 2023 and 2024, respectively.

Archived SPATT extractions were selected before (*n* = 1), during (*n* = 3), and after (*n* = 1) each toxin event to compare environmental metabolomics (Figure 1B). Positive mode ionization detected 3578 total features, and negative mode detected 3781 features (Figure 4A). The *m*/*z* and ion intensity range were comparable (Table 2). Features detected in samples from both years were included in a principal component analysis. Approximately half of the variance was explained on PC 1 and PC2 for both positive (49.9%) and negative (53.0%) mode data (Figure 4B and Figure S2). The two events separated on PC 1, indicating greater variation between years than between samples within a single year. However, the first time point of the 2023 event and the last time point from the 2024 event are the least differentiated (Figure 4B). This result may be related to the temporal overlap of the SPATT samplers, with the first sample of 2023 occurring in March and the last sample of 2024 occurring in April (Figure 1B). This pattern suggests that PC 1 is separating the events by seasonality, rather than simply because they occurred in different years, but there is not a clear delineation of sampling time in the PCA.

The top 5 features with the highest contribution to PC1 are shown in Figure 4C,D though it should be noted that the summed contribution was only 0.43% and 0.34% for positive and negative mode, respectively (Figure S2). In positive mode, this included 3 features that were part of large molecular families, a pair, and a singleton, based on GNPS spectral clustering algorithms (Figure 4C). The networks including these features generally had a large proportion of nodes that varied in ion intensity between 2023 and 2024, shown by pie charts in Figure 4. The top contributor had a mean intensity of 19071.6. Negative mode did not show the same degree of connectivity with 2 pairs and 3 singletons, and the variation between 2023 and 2024 samples was less pronounced (Figure 4D).

**Figure 5:**
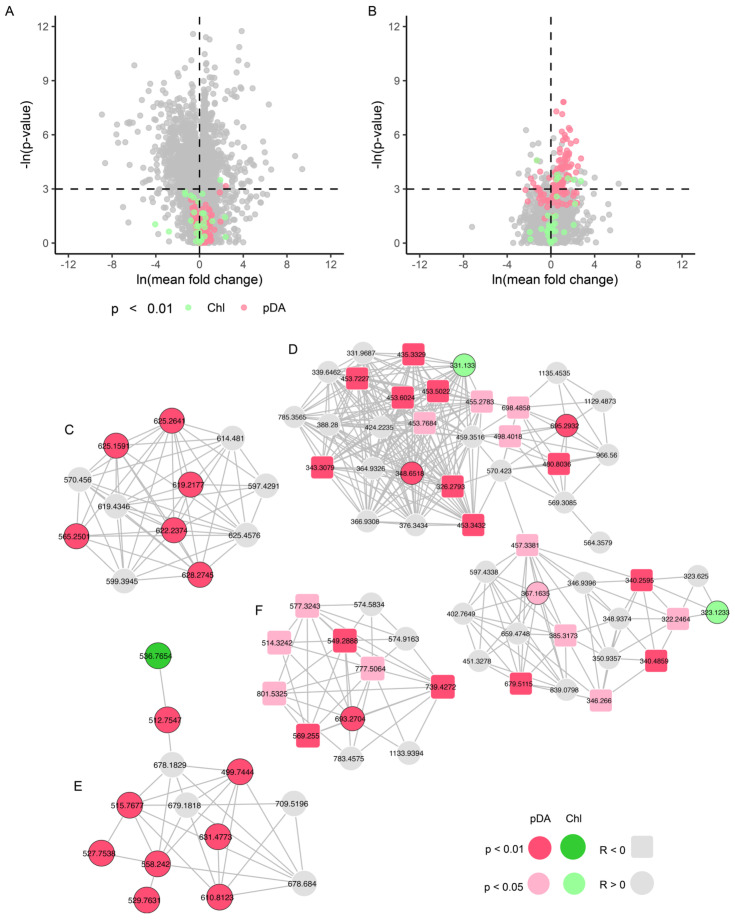
Volcano plots with mean fold change and *p*-values (**A**) for all dates in 2023 vs. 2024 and (**B**) all dates without DA vs. dates with DA. (**C**,**D**) Positive mode and (**E**,**F**) Negative mode molecular families with features that are correlated to pDA and Chl.

The features were then compared across two sets of categorical variables (1) Year (2023 vs. 2024) and (2) Event (Time points 1 and 5 vs. time points 2, 3, and 4). Consistent with results from the PCA, 31% of features experienced a significant mean fold change (*p* < 0.05) between 2023 and 2024. Only two of these features were correlated to Chl or DA (Spearman ρ, *p* < 0.01), and both were elevated in 2024 (Figure 5A). In the Event comparison, 97% of features did not change significantly. Of the 3% showing significant change, 68 were elevated during the toxin event and correlated to DA and 7 were reduced and negatively correlated to DA (Spearman ρ, *p* < 0.01, Figure 5B). Raising the correlation threshold to 0.05, gave 159 pDA-correlated features with 109 features significantly elevated or reduced in the event analysis. At both thresholds in the event analysis, pDA-correlated features were 5–10 times more numerous than Chl-correlated features, suggesting that pDA or *Pseudo-nitzschia* was a stronger driver for differences in the environmental metabolome across the toxin events. Both ESI modes showed molecular families with multiple nodes that were significantly correlated to pDA, suggesting shared structural similarities and intensity patterns during both toxin events (Figure 5C–F). Clustering within molecular families was also observed for Chl (Figure S3). However, we could not identify any library matches to features related to pDA or Chl.

**Figure 6:**
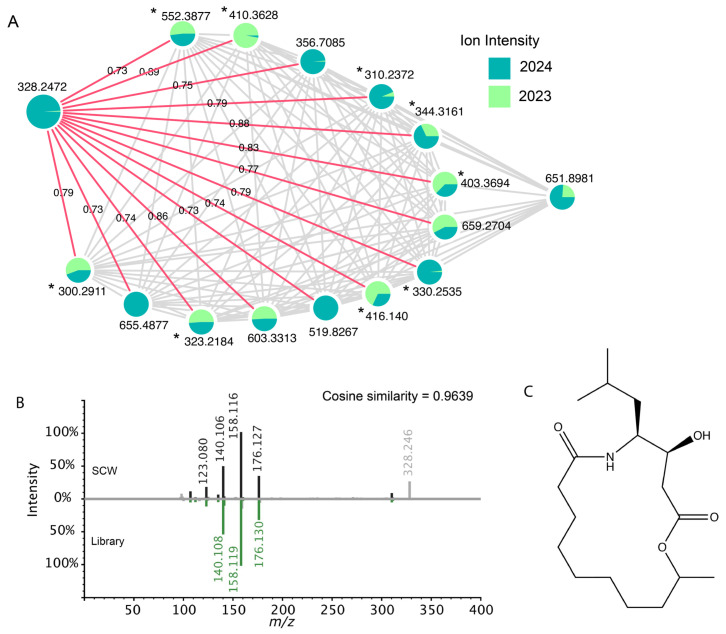
(**A**) Molecular family including cabrillostatin. Pie charts represent the mean intensity for 2023 vs. 2024, and analogues are marked with *. Nearest neighbor edges are labeled with cosine similarity scores. (**B**) Mirror match between *m*/*z* 328.2473 from SCW (**top**) and cabrillostatin library spectrum (**bottom**). (**C**) Molecular structure of cabrillostatin.

Overall, feature-based molecular networking in GNPS returned 7 unique library hits and 200 unique analogues in positive mode, and 1 library hit and 52 analogues in negative mode. One of the library matches in positive mode was cabrillostatin, which was first characterized in HP20 deployments from Southern California (Figure 6) [32]. Our spectra show *m*/*z* 328.2472 [M+H]^+^, 5 shared peaks with the library reference, and 0.9 cosine similarity (Figure 6B). The molecular family including cabrillostatin had 15 nodes with a high degree of interconnectivity. Except for *m*/*z* 651.8981, all nodes in the cluster were related to cabrillostatin at a cosine threshold of 0.7 (Figure 6A). Eight of the features in the cluster were identified as potential analogues of cabrillostatin (Figure 6A). Cabrillostatin and five neighboring nodes were primarily detected in samples from 2024. These features had a similar temporal pattern, with ion intensity peaking in samples from 13 March 2024 (Figure S4). In addition, *m*/*z* 328.2472 was by far the most pronounced compound in the network, ion intensity was approximately 20 times greater than the next feature, *m*/*z* 655.4877 (Figure S4).

In-silico dereplication was also performed using Progenesis QI theoretical fragmentation algorithms on the Natural Products Atlas. This method found 6269 potential identifications for 1999 unique features with scores ranging from 18–55.5. Limiting the results to identifications with a score greater than or equal to 50, returned 103 potential identifications for 26 features (Figure S5A). While additional structure elucidation work is needed, the highest scoring identifications mostly originated from fungi, including genera *Ganoderma*, *Antrodia*, and *Aspergillus*. Identifications with bacterial origins included the genera *Streptomyces* spp., *Thermosynechococcus* spp., and *Anabaena* spp. (Figure S5B). Two features had identification scores greater than 50 and were positively correlated to pDA (*p* < 0.01), Vicenistatin M from *Streptomyces* bacteria and Serinocyclin A from *Metarhizium* fungus (Figure S6).

## 3. Discussion

DA events at the SCW generally occur during spring and summer when upwelling intensity coincides with spring bloom conditions, such as increased day length and warmer air temperature. Specific drivers of these events are unknown, posing a key challenge for HAB prediction and toxin mitigation. Furthermore, the plasticity of toxin production and phytoplankton succession coupled with our lack of understanding for the ecological role of DA, adds additional complexity. While the present study is not inclusive of all hypothesized HAB drivers, we summarize key aspects of the physical, biological, and chemical conditions coinciding with elevated pDA. The study leveraged co-located sampling modalities from the SCW and Monterey Bay region during two springtime pDA events. Physical dynamics, phytoplankton observations, and environmental metabolomics via SPATT passive samplers showed how comparable pDA events may emerge from distinct environmental conditions.

Physical drivers of pDA in the CCS have been previously attributed to upwelling, which is strongly related to climate patterns, surface temperature, and nutrients [19]. The most destructive *Pseudo-nitzschia* HAB on the Pacific West Coast in recent history happened during the Northeast Pacific Warm anomaly in 2015–2016. *P. australis*, a highly toxic species, extended north of its native range [21]. The onset of the upwelling season supplied nutrients, while El Niño storms advected offshore populations toward the coast [14,33]. The extreme event has been associated with high cDA, ocean warming, and storm activity [33]. Our results implicate environmental dynamics akin to the 2015 extreme event in smaller pDA events at the SCW as well, suggesting continued likelihood of future extreme toxin events in this region. Comparing DA events in 2023 and 2024 is also useful for understanding why toxins from HABs are difficult to predict from seasonality alone.

We propose two potential mechanisms for the 2024 DA event: (1) the advection of a highly toxic, offshore population of *P. australis* and (2) the influx of terrestrial nutrients from a series of winter storms related to El Niño. In 2024, pDA peaked before the spring bloom, which occurred in May and was dominated by *Eucampia* spp. diatoms. As previously described, upwelling favorable winds drive the formation of the upwelling shadow at SCW. In contrast, downwelling favorable winds increase the connectivity between the sample site and surrounding regions [28,34]. Downwelling and shoreward advection have also been linked to coastal DA events in Washington and Oregon [35,36]. While advected surface waters may be nutrient deficient, they are generally warmer, conditions which have been shown to induce DA in culture experiments [14]. In addition, a study from Monterey Bay in 2018 found highly toxic *P. australis* made up a large proportion of the offshore community, while *Pseudo-nitzschia fradulenta*, which is relatively less toxic, dominated areas closer to the coast [37]. Advection could have seeded the SCW site with an offshore *P. australis* strain in the 2024 event. As a proportion of total Chl, *P. multiseries* from the spring bloom in May was 4 times more abundant than *P. australis* from the toxin event in March, but only minimal DA was detected. This significant shift highlights that brief establishment of a highly toxic population can have a large relative impact on DA production in the environment. However, as *P. australis* was implicated in both toxin events, drivers for the large difference in cDA are still unclear. A metagenomic analysis would be a valuable complement to the present study, enabling genetic differentiation of the two *Pseudo-nitzschia* populations [21].

El Niño is related to increased precipitation and winter storm activity on the California west coast, which in turn, effects Monterey Bay through freshwater influx of dissolved organic matter and nutrients [18]. Submarine ground water discharge is also a potentially significant nutrient source during periods of high precipitation [38]. These influxes may compensate for the reduction in upwelled nutrients supplying the coast during El Niño. A series of storms in the winter of 2024 increased San Lorenzo River discharge less than 1 km from the sample site. In fact, storm activity on Feb 19, caused a local flow maximum 2 days before pDA was detected (Figure S7). In opposition, a previous logistic modeling study at the SCW found periods of downwelling and high river discharge suppressed the formation of *Pseudo-nitzschia* HABs [39], and decreasing salinity has also been found to negatively affect toxicity [40,41]. Additional data on source nitrogen in surface waters during the 2024 event would help elucidate contributions of terrestrial nutrients to the study area.

The 2023 event was driven by the upwelling season and spring bloom environment. There was a strong correlation between the 2023 event and upwelling, offshore temperatures were cold, and BEUTI suggested a supply of deep nitrate to surface waters. Finally, the divergence of temperature between SCW and offshore buoy is characteristic of upwelling shadow development, which occurs during sustained upwelling in the CCS. The Monterey Bay upwelling shadow has been proposed as a significant bloom incubator for dinoflagellates due to increased stratification, poor surface nutrients, and warm conditions [42,43]. However, many of these same conditions, especially silica and phosphate limitation, have also been shown to induce DA production in toxigenic *Pseudo-nitzschia* [40,44]. In the 2023 event, the onset of the upwelling shadow may have favored *P. australis*. After the bloom peak, we observed a rapid shift to dinoflagellates in the phytoplankton community. This could be explained if the nutrient supply became too limited to sustain high diatom biomass, so dinoflagellates, which have the ability to migrate to the nitracline at night, gained a competitive advantage [45].

The ecological role of DA is still largely unknown, and toxin is not often well correlated to phytoplankton concentration, as observed during the 2024 spring bloom. There is strong evidence that DA production is regulated by allelopathy [4]. Multiple studies measure increased DA in response to grazer cues, suggesting its role as a chemical defense [24,46,47]. There is also a rapidly growing body of work on inter-specific associations between diatoms and bacteria [4]. These interactions may be parasitic, competitive, or synergistic and involve the exchange of organic metabolites, vitamins, and trace metals [48]. *Pseudo-nitzschia* microbiomes are highly specific [49], and inclusion of bacteria to axenic cultures effects DA production [50] and growth rates [51]. Though the mechanism for bacterial regulation of DA is still unknown, toxic and non-toxic *Pseudo-nitzschia* populations have distinct microbiomes [25], and associated bacteria may provide the host cell with pre-cursor metabolites for the DA biosynthesis pathway [49,52].

Untargeted metabolomics uses mass spectrometry to identify known and unknown chemical features in a sample. Applied to marine microbial communities, untargeted approaches can generate a wealth of information about the production of dissolved metabolites, leading to new theories of ecological function [53]. The present study employed SPATT passive samplers and untargeted metabolomics pipelines to observe changes in the chemical environment across DA events. Consistent with a previous study using SPATT to create “chemical profiles” of HAB events [54], chemical diversity at the SCW differed between timepoints. In addition, interannual variation was greater than intra-annual variation. Most features were detected during both years, and there was a large variation in intensity. While we could not confirm the identity of features with strong correlations to pDA, a passive sampling approach shows promise for unraveling in situ microbial interactions. Future work would benefit from increasing temporal sampling resolution to capture real-time shifts in chemical and biological environments. Finally, the SPATT dataset included potential identifications to metabolites from freshwater cyanobacteria (Figure S8). Influx of cyanobacterial toxins to coastal marine sites presents a new threat frontier for HAB monitoring and could be explored further with untargeted mass spectrometry and SPATT [55].

Feature-based molecular networking (FBMN) within GNPS was used to dereplicate unknowns and visualize molecular families [56,57] from SPATT extracts. FBMN builds on classical molecular networking by improving feature quantification and isomer differentiation [56]. Our results show a putative identification of cabrillostatin, which was first characterized in HP20 field samplers from the Cabrillo Marine Reserve in Southern California [32]. Cabrillostatin appears to be an under-reported but ubiquitous component of dissolved organic matter and was identified in 11 datasets of nearshore and deep dissolved organic matter [32]. The present study’s detection of cabrillostatin and analogues, lends additional proof-of-concept to diverse applications of HP20 SPATT in environmental monitoring. Molecular networking and HP20 passive sampling has also been presented as an novel approach to drug discovery via marine natural products [32]. In the same vein, environmental metabolomics applied to HAB monitoring takes a “compound-first” perspective on metabolites related to toxin production, which may shed light on new potential functions for DA in the marine microbial community [32].

## 4. Conclusions

At first glance, two spring DA events occurring in consecutive years at the same site share a resemblance. However, deeper analysis shows that different environmental conditions gave rise to each event. This study included public, historical data from the SCW and other regional sites to compare physical dynamics and community structure during each toxin event. We also conducted an opportunistic analysis of environmental metabolomics from archived SPATT samples across both toxin periods. The event in 2023 was consistent with traditional spring bloom conditions and *Pseudo-nitzschia* was the dominant taxa. The following year, pDA increased approximately two months ahead of the spring bloom, driven by anomalous conditions from El Niño and warmer temperatures. During the 2024 event, *Pseudo-nitzschia* was not the dominant taxa and biomass was relatively low, implying that the 2024 population of *P. australis* was about two orders of magnitude more toxic than 2023. A novel application for SPATT was used to profile shifts in the chemical environment. We detected 159 metabolites that were strongly correlated with DA across both toxin events and reported a putative identification for cabrillostatin, a recently discovered marine natural product, for the first time in Monterey Bay. The results highlight the challenge of univariate-based toxin predictions and encourage ongoing development of environmental metabolomics in HAB monitoring.

## 5. Materials and Methods

### 5.1. Online Data Acquisition

**Figure 7:**
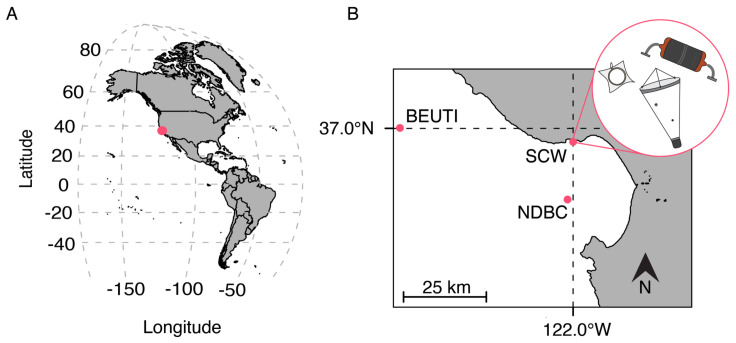
(**A**) Monterey Bay, California and (**B**) Santa Cruz Wharf (SCW) with co-located sampling methods, NDBC 46092, and 37° N for estimating the biologically effective upwelling transport index (BEUTI).

Data for offshore temperature and wind was retrieved from the National Data Buoy Center 46092 (36.75° N, 122.03° W) from February–June in 2023 and 2024 (Figure 7). Alongshore winds were rotated with respect to the California coastline before analysis. Nitrate flux from upwelling was estimated using the Biologically Effective Upwelling Transport Index (BEUTI) at 37° N [58]. Co-located biological and temperature data was acquired at the Santa Cruz Municipal Wharf (SCW, 36.9° N, 122.0° W, Figure 7). The SCW dataset includes weekly measurements of pDA (µg L^−1^), Chl (mg m^−3^), *Psuedo nitzschia* spp. abundance (cells L^−1^), and temperature (°C). Data is publicly available at https://erddap.sccoos.org/erddap/tabledap/HABs-SantaCruzWharf.subset (accessed 24 August 2024). Briefly, samples are collected approximately 300 m from shore with 8 m mean water depth and 2.7 m maximum tidal range. Seawater was collected in a Van Dorn sampler at 3 discrete depths (0 m, 1.5 m, 3 m) and combined as an integrated depth sample. Whole water was filtered by GF/F (Whatman) for pDA and Chl analysis. Abundance for *Pseudo-nitzschia australis* and *Pseudo-nitzschia multiseries* were enumerated from 10 mL of seawater using large subunit rRNA probes and methods described in [59] The total cell concentration of both species is calculated as a ‘seriata size class’ as described in [37] Additional sampling details found in [28]. Relative abundance index (RAI) and water color was retrieved from the SCW plankton blog (https://phytoblog.sites.ucsc.edu, accessed 20 February 2025). The plankton blog is a searchable resource containing more than a decade of weekly phytoplankton observations at two sites in Monterey Bay. Samples used to calculate RAI, were collected by vertically towing a plankton net (20 µM, 25 × 90 cm) 5 times through 3 m depth and evaluated under stereo microscope (Leica). RAI designations are: Abundant (>= 50%), Common (10–49%), Present (1–9%), Rare (<1%).

### 5.2. SPATT Sampling

Opportunistic, archived SPATT samples were selected for passive chemical sampling during both toxin events. Sample dates chosen across the events in 2023 and 2024, including one pre-event sample, 3 samples during elevated pDA, and a post-event sample (Figure 1). Damage to the SCW during a storm in February 2024, reduced SPATT coverage during the initial phase of the 2024 toxin event. SPATT samplers were constructed by enclosing Diaion^®^ HP20 resin (3g, Sigma-Aldrich, St. Louis, USA) between two pieces of nylon mesh and securing the mesh in a plastic embroidery hoop [60]. SPATT was activated by soaking in 100% MeOH, rinsed thoroughly in Milli-Q water (MQ), and deployed at the SCW at approximately 2.5 m depth for 7 days. Upon retrieval, SPATT was rinsed once in MQ and stored at −80 °C until extraction. SPATT was then thawed and rinsed briefly in deionized water to remove debris from the mesh. Two method blanks per year were prepared by constructing the SPATT ring, activating in 100% MeOH, and rinsing in MQ. For samples and blanks, the resin was scraped into empty polypropylene columns (Bio Rad, Hercules, USA, Econo-Pac^®^ 732-1010) attached to a vacuum manifold, desalted with MQ and vacuumed until dry. Extracts were eluted dropwise with 4 × 5 mL of 100% MeOH. The final extract was vortexed, and 1 mL transferred to an LCMS vial for analysis.

### 5.3. Untargeted LCMS/MS

Untargeted analysis of SPATT extracts was performed on a Waters XEVO™ G3 Q-TOF mass spectrometer (Waters Corporation, Milford, USA) with a Waters ACQUITY I-Class Plus (Waters Corporation, Milford, MA, USA). Liquid chromatography used column HSS T3 2.1 × 100 mm at 65 °C with 5 µL injection. Elution involved a gradient of Solvent A: 0.1% aqueous formic acid and Solvent B: 0.1% formic acid in acetonitrile (ACN, Table S2). All reagents were LCMS grade. Data were collected in positive and negative electrospray ionization (ESI) modes. Mass spectrometry parameters included 1.2 kV ESI capillary voltage, 30 V cone voltage, 1000 LPH desolvation gas, 450 °C desolvation temperature, and 0.3 s accumulation time. The mass range was set from 95–1200 Da. Two function low energy was 6 V and high energy ramped from 20–45 V. The time of flight (TOF) analyzer was tuned to 25,000 resolution at *m*/*z* 500 and leucine enkephalin lock mass used LockSpray 1 accumulation per 30 s [61].

Features were initially filtered using Progenesis QI (Waters Corporation, Milford, MA, USA). After alignment and data normalization, peaks were rejected if they had a high coefficient of variation between samples (CV > 30%) or a peak width greater than 1 min. Then ANOVA (*p* = 0.05) was calculated between blanks and sample groups. Components observed in both groups did not pass the significance test and were excluded from further analyses.

### 5.4. Dereplication and Molecular Networking

Chemical features exported from Progenesis QI were queried against bacterial and fungal products in the Natural Product Atlas 2.0 (NPA) using the theoretical fragmentation algorithm in Progenesis QI [62,63]. The exact mass and fragment tolerance threshold was 10 ppm. For multiple NPA matches per feature and a total identification score above 50, we reported the compound with the maximum fragmentation score.

Positive and negative mode feature-based molecular networking was completed using the Global Natural Products Social Molecular Networking (GNPS) online platform (https://gnps.ucsd.edu, accessed 13 April 2025) [57]. MS /MS fragment ions within ±17 Da of the precursor were filtered out, and MS/MS spectra were window filtered by choosing only the top 6 fragment ions in the ±50 Da window throughout the spectrum. Precursor ion mass tolerance and MS/MS fragment ion tolerance were both set to 0.05 Da. Edges of the network were filtered to have a cosine score above 0.7 and at least 4 matched peaks. Edges were retained between two nodes if each of the nodes appeared in each other’s respective top 20 most similar nodes. The maximum molecular family was set to 200 by removing the lowest scoring edges. Analogues were also searched against MS/MS spectra with a maximum difference of 100.0 in the precursor ion value and the same library search parameters of a score above 0.7 and at least 4 matched peaks. Molecular networks created in GNPS were visualized using Cytoscape software (Version 3.10.3) [64].

### 5.5. Statistics and Visualization

R studio was used for statistics and data visualization [65]. A toxin event was designated when two consecutive sampling dates had detectable levels of pDA. The event duration was the number of days between the first sampling date of a toxin event and the sampling date prior to pDA receding below the limit of detection. Daily means for wind and water temperature were calculated from hourly data from NDBC 46092, and temperature anomaly was calculated from mean daily temperatures across 37 years (1987–2024). Prior to principal component analysis, data for winds, temperature, and BEUTI were zero-mean scaled. PCA used FactoExtra (Version 1.0.7) and FactoMineR (Version 2.11) packages [66,67].

The relative abundance index (RAI) was visualized by applying a score to each taxa, corresponding to the maximum range of its RAI designation (R = 1, P = 9, C = 49, A =100). Summary pie charts were generated by defining a pre-bloom period (35 days) before the toxin event and a post-bloom period (35 days) after the toxin event for both years. RAI scores from 20 most abundant taxa were used to find the proportion of each functional group (dinoflagellate, diatom [excluding *Pseudo-nitzschia*], and *Pseudo-nitzschia*) and RAI category that fell into each period. cDA was calculated by dividing the pDA concentration by the total *Pseudo-nitzschia* sp. concentration for each sampling date in the toxin event.

For LCMS/MS data, 3 technical replicates were used to calculate the normalized mean ion intensity. The mean intensity for each feature on each sample date was zero-mean scaled prior to PCA using the FactoExtra package in R [66]. Volcano plots of mean fold change vs. *p*-value (Welch’s *t*-test) were generated by binning timepoints by (1) 2023 (*n* = 5) vs. 2024 (*n* = 5) and (2) Toxin event (*n* = 6) vs. non-event (*n* = 4). Prior to Spearman rank correlation coefficients, SCW pDA and Chl were linearly interpolated to obtain matching sampling dates with SPATT. Only features that were detected in both years were included in PCA, volcano plots, and correlation analyses.

## Figures and Tables

**Table 1:** Summary of toxin events in 2023 and 2024 with a comparison to significant historical events at the same site. The event mean shows the mean concentration for all samples within the event period.

Toxin Event	Event Mean (µg L^−1^)	Event Duration (Days)
April–May 2023	0.16	35
February–March 2024	0.12	35
March–June 2014	2.41 (Max)	85
May–September 2016	0.73	133 (Max)
Mean	0.29	27

**Table 2:** Overview of the number of features detected by untargeted LCMS/MS in both positive and negative ESI modes.

ESI 2023	Only 2023	Only 2024	Both Years	*m*/*z* Range	Intensity
Positive	148	161	3269	300−1195	1.3−183,267.6
Negative	42	93	3646	300−1199	0.2−1,888,801.9
Total			6915		

## Data Availability

The original contributions presented in this study are included in the article/supplementary material. Access negative mode GNPS Feature-based molecular networking is at https://gnps.ucsd.edu/ProteoSAFe/status.jsp?task=cfeb09b9683f4b1b8125cbc5d4f10acb and positive mode at https://gnps.ucsd.edu/ProteoSAFe/status.jsp?task=462fe0b422aa47189cc634f9c98655e4. Further inquiries can be directed to the corresponding author(s).

## Supplementary Materials

The following supporting information can be downloaded at: https://www.mdpi.com/article/10.3390/toxins17100511/s1, Table S1: The record of toxin events at the Santa Cruz Wharf. A toxin event is defined when domoic acid was detected for at least 2 consecutive weeks. Units: Duration (Days), pDA (µg DA L^−1^), Mean & Sum (µg DA mL^−1^); Table S2: LC gradient parameters for positive and negative mode untargeted analysis; Figure S1: (A) Color tile values during winter and spring of 2023 and 2024. (B) Count of each tile number during the study period; Figure S2: Principal component analysis statistics for (A) positive and (B) negative mode features detected during 2023 and 2024; Figure S3: (A) Positive and (B) Negative mode molecular networks from GNPS. Correlation to pDA (pink) or Chl (green) at *p* < 0.05 (light) and *p* < 0.01 (dark). Nodes are labeled with precursor mass; Figure S4: Cabrillostatin cluster mean ion intensity over time; Figure S5: (A) Potential identifications from Progenesis QI fragmentation algorithm and the Natural Product Atlas. (B) Highest scoring genera from Bacteria and Fungi; Figure S6: Two features (A) *m*/*z* 524.33 and (B) *m*/*z* 695.29 with positive correlations to pDA were identified by Progenesis QI via theoretical fragmentation and the Natural Products Atlas. Yearly scaled SPATT detection, Progenesis QI fragment match, summary information, and chemical structure for potential match with bacterial compound, Vicenistatin M. Blue rectangle = 2024 event, green rectangle = 2023 event; Figure S7: Flood gauge data from NOAA and USGS were used to observe freshwater discharge to the Santa Cruz Wharf from the San Lorenzo River; Figure S8: Potential matches from SPATT extracts to natural products from cyanobacteria.

## Abbreviations

The following abbreviations are used in this manuscript:

ACN	Acetonitrile
ASP	Amnesiac shellfish poisoning
BEUTI	Biologically effective upwelling transport index
CCS	California current system
Chl	Chlorophyll
cDA	Cellular domoic acid
DA	Domoic acid
ENSO	El Niño Southern Oscillation
ESI	Electrospray ionization
FBMN	Feature based molecular networking
GF/F	Glass fiber filter
GNPS	Global natural products social molecular networking
HABs	Harmful algal blooms
LCMS/MS	Liquid chromotagraphy tandem mass spectrometry
MeOH	Methanol
MQ	Milli-Q water
NDBC	National data buoy center
NPA	Natural product atlas
PCA	Principal component analysis
pDA	Particulate domoic acid
RAI	Relative abundant index
SCW	Santa Cruz Municipal Wharf
SPATT	Solid-phase adsorption toxin tracking
