# Supplementary material for: One Bloom Is Not Like the Other—Distinct Environmental Drivers Result in Domoic Acid Events in Monterey Bay, California
Source: Toxins (Basel). 2025 Oct 17;17(10):511. doi: 10.3390/toxins17100511 (PMC12567695; doi:10.3390/toxins17100511)
Supplement: Supplementary file 1 [file toxins-17-00511-s001.zip › toxins-3899917-supplementary.pdf]

Table S1. The record of toxin events at the Santa Cruz Wharf. A toxin event is defined when domoic acid was detected for at least 2 consecutive weeks. Units: Duration (Days), pDA (ng DA L^{-1}), Mean & Sum (ng DA mL^{-1}).

Event	Start	End	Duration	pDA	Mean	Sum
1	10/5/11	11/9/11	35.0	0.915077, 0.060326, 0.261097, 0.052310, 0.046524, 3.814660	858.332	5149.994
2	11/23/11	12/14/11	21.0	3.127740, 0.797935, 0.090507, 0.540992	1139.294	4557.174
3	4/18/12	6/12/12	55.0	0.012838, 0.081947, 0.476503, 0.020382, 0.231761, 0.171555, 0.357468, 0.161597, 0.059673	174.858	1573.724
4	6/27/12	7/11/12	14.0	0.016587, 0.018632, 0.040977	25.399	76.196
5	7/26/12	8/29/12	34.0	0.007929, 0.514852, 1.694617, 9.356503, 2.768953, 0.009041	2391.923	14351.895
6	9/19/12	9/26/12	7.0	0.059437, 0.007219	33.328	66.656
7	10/24/12	11/21/12	28.0	0.026463, 0.109445, 0.015739, 0.018306, 0.064967	46.984	234.920
8	3/12/14	6/4/14	84.0	0.087780, 0.143520, 1.684400, 0.803640, 0.666160, 0.669580, 0.091920, 1.233220, 5.554738, 3.435198, 0.150000, 0.592932, 16.220020	2410.239	31333.108
9	7/9/14	7/16/14	7.0	0.035462, 0.123398	79.430	158.860
10	4/1/15	7/8/15	98.0	0.004080, 0.018740, 0.008840, 0.622140, 3.422408, 3.179484, 0.311960, 3.874320, 6.630000, 0.056900, 0.539175, 0.823639, 0.572427, 0.156511, 0.024049	1349.645	20244.673
11	7/29/15	8/5/15	7.0	0.081306, 0.289930	185.618	371.236
12	9/2/15	9/23/15	21.0	0.017950, 0.035560, 0.512930, 0.052370	154.703	618.810

13	11/18/15	11/25/15	7.0	0.008832, 0.013347	11.090	22.179
14	5/11/16	9/21/16	133.0	0.022350, 0.029610, 0.038580, 0.107480, 0.176630, 0.734420, 0.027520, 0.558830, 1.275660, 2.437170, 5.804060, 1.110750, 0.354560, 0.109330, 0.114447, 0.092984, 0.219574, 0.374587, 0.945935, 0.029048,	728.176	14563.525
15	10/12/16	10/19/16	7.0	0.012923, 0.015278	14.101	28.201
16	11/16/16	12/7/16	21.0	0.000046, 0.000048, 0.000051, 0.000032	0.044	0.177
17	3/29/17	4/19/17	21.0	0.011194, 0.024891, 0.012771, 0.341472	97.582	390.328
18	5/24/17	6/28/17	35.0	0.091944, 0.069768, 0.727049, 0.620082, 1.158715, 0.545994, 0.321019	504.939	3534.571
19	1/31/18	3/14/18	42.0	0.234962, 0.051417, 0.004071, 0.164432, 0.006546, 0.011502	78.822	472.930
20	3/28/18	4/18/18	21.0	0.008825, 0.031029, 0.095734, 0.083973	54.890	219.561
21	5/16/18	6/13/18	28.0	0.003717, 0.029955, 0.071442, 0.519268, 0.099427	144.762	723.809
22	6/28/18	8/15/18	48.0	0.049075, 0.023815, 0.832841, 0.787071, 0.022102, 0.019747, 0.047559, 0.140234	240.306	1922.444
23	10/31/18	11/7/18	7.0	0.012863, 0.034381	23.622	47.244
24	12/19/18	1/9/19	21.0	0.025659, 0.016606	21.132	42.265
25	5/29/19	6/5/19	7.0	0.175411000, 0.025936000	100.674	201.347
26	10/9/19	10/30/19	21.0	0.045320000, 0.035235000, 0.017155000, 0.003418000	25.282	101.128
27	2/12/20	3/4/20	21.0	0.024307000, 0.027913000, 0.285985000, 0.025100000	90.826	363.305
28	5/5/20	6/16/20	42.0	0.022261000, 0.146040000, 0.787506000, 0.014500000, 6.120819000, 0.014732000, 0.007263000	1016.160	7113.121

29	6/30/20	7/21/20	21.0	0.148906000, 0.005920000, 0.007751000, 0.007593000	42.542	170.170
30	8/18/20	9/15/20	28.0	0.010073000, 0.295338000, 0.137262000, 0.028699000	117.843	471.372
31	10/6/20	10/13/20	7.0	0.004589000, 0.008345000	6.467	12.934
32	11/17/20	12/15/20	28.0	0.006683000, 0.070168000, 0.003430000, 0.002491000, 0.003830000	17.320	86.602
33	2/24/21	3/3/21	7.0	0.303795000, 0.232340000	268.068	536.135
34	11/24/21	1/5/22	42.0	0.005857490, 0.002824091, 0.001176530, 0.014590840, 0.003613496, 0.021348195	8.235	49.411
35	1/19/22	2/2/22	14.0	0.001361654, 0.005852328, 0.001740605	2.985	8.955
36	3/9/22	4/20/22	42.0	0.003059287, 0.065133000, 0.031029000, 0.015763000, 0.005996000, 0.001180000, 0.029874000	21.719	152.034
37	6/29/22	7/6/22	7.0	0.005513000, 0.008843000	7.178	14.356
38	1/25/23	2/1/23	7.0	0.003204000, 0.005127000	4.165	8.331
39	4/19/23	5/24/23	35.0	0.038201000, 0.107839000, 0.354176000, 0.119062000, 0.300577000, 0.010667112	155.087	930.522
40	8/2/23	8/23/23	21.0	0.002981000, 0.002336000, 0.004497000, 0.002563000	3.094	12.377
41	10/11/23	10/18/23	7.0	0.003449000, 0.013677000	8.563	17.126
42	2/21/24	3/27/24	35.0	0.023836000, 0.157651000, 0.331107000, 0.080481000, 0.123292000, 0.026366000	123.788	742.733
43	5/8/24	5/15/24	7.0	0.049856000, 0.010593655	30.225	60.450
44	6/12/24	6/19/24	7.0	0.015362000, 0.015362000	15.362	30.724
**Mean**			**27.5**		**291.701**	**2540.628**

Table S2. LC gradient parameters for positive and negative mode untargeted analysis.

Time (min)	Flow (mL min^{-1})	A (%)	B (%)
Initial	0.3	98	2
0.5	0.3	98	2
7	0.3	1	99
8.75	0.3	1	99
8.76	0.3	98	2
10	0.3	98	2

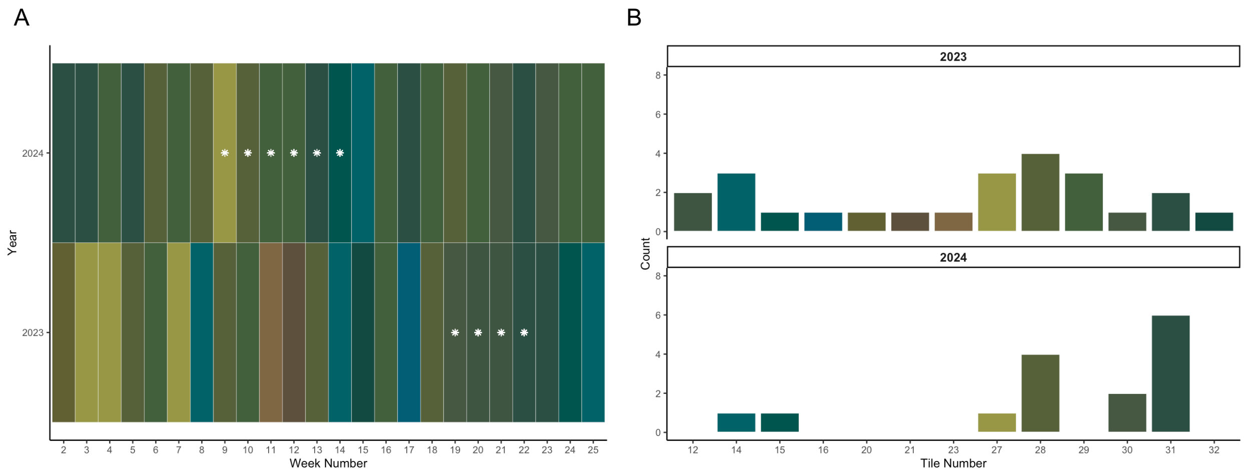

Figure S1. A) Color tile values during winter and spring of 2023 and 2024. B) Count of each tile number during the study period. Tile data acquired from the Monterey Plankton Blog (https://phytoblog.sites.ucsc.edu/). Additional information about the "What Color is Your Water?" project can be accessed at (http://oceandatacenter.ucsc.edu/PhytoBlog/color.html). *Indicates weeks of toxin event.

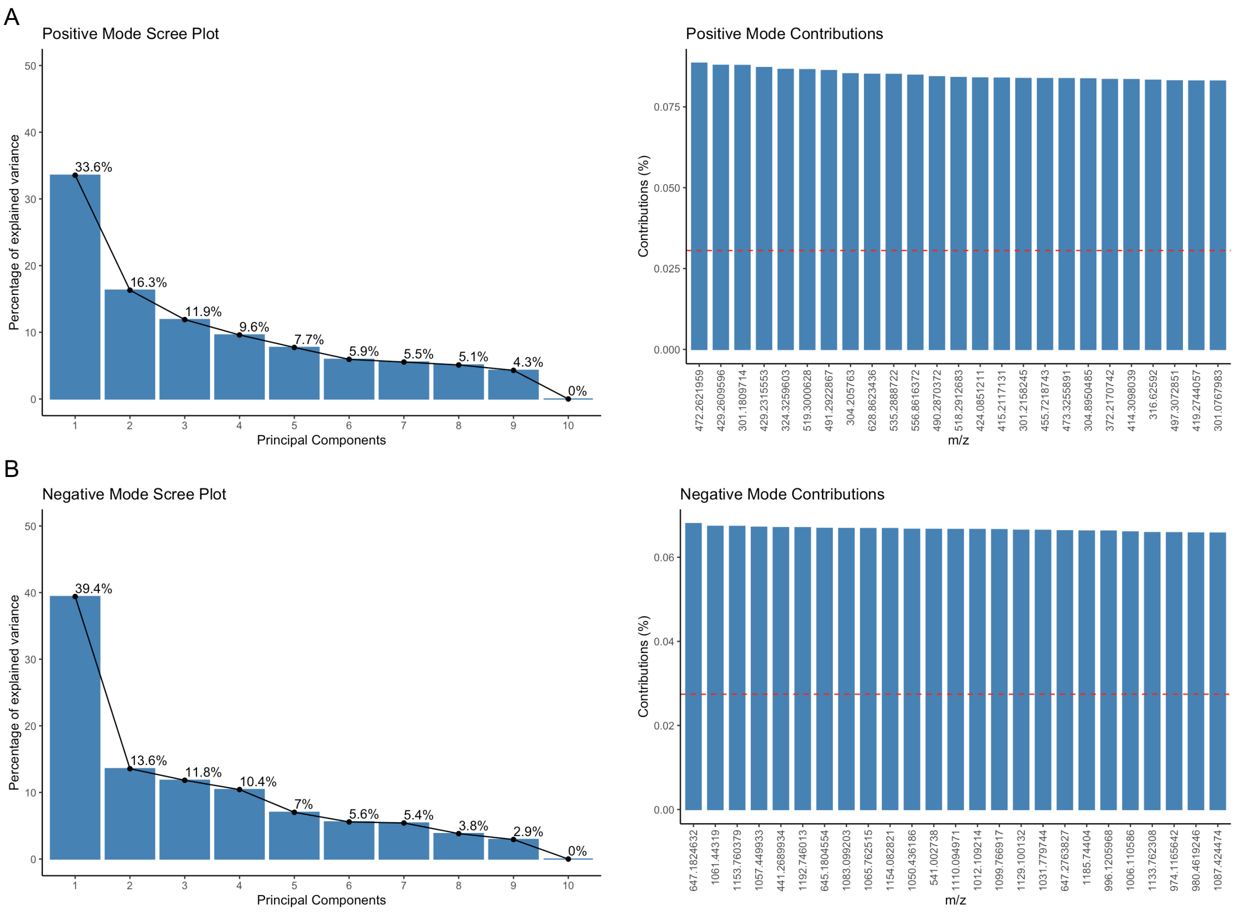

Figure S2. Principal component analysis statistics for A) positive and B) negative mode features detected during 2023 and 2024.

A

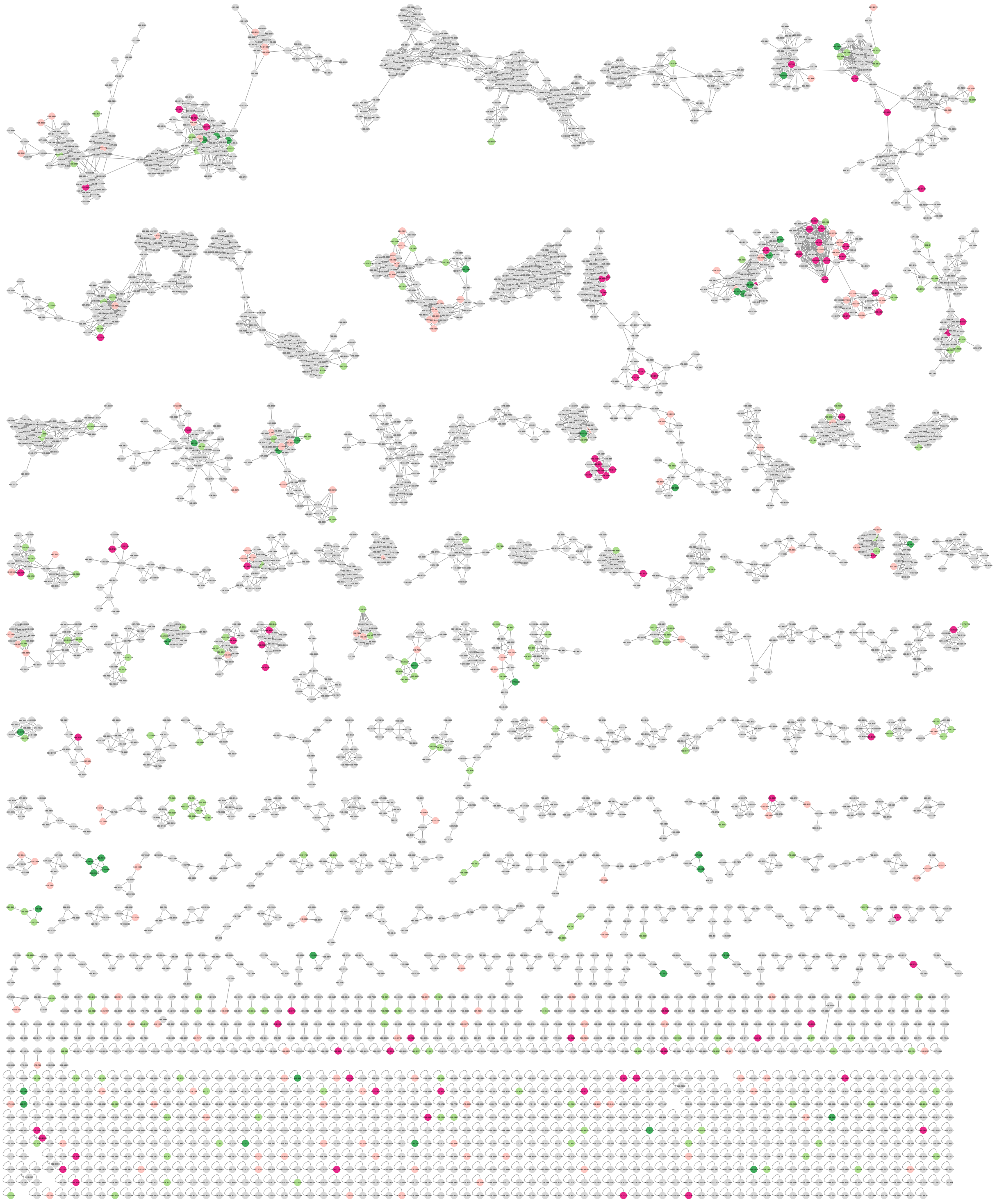

B

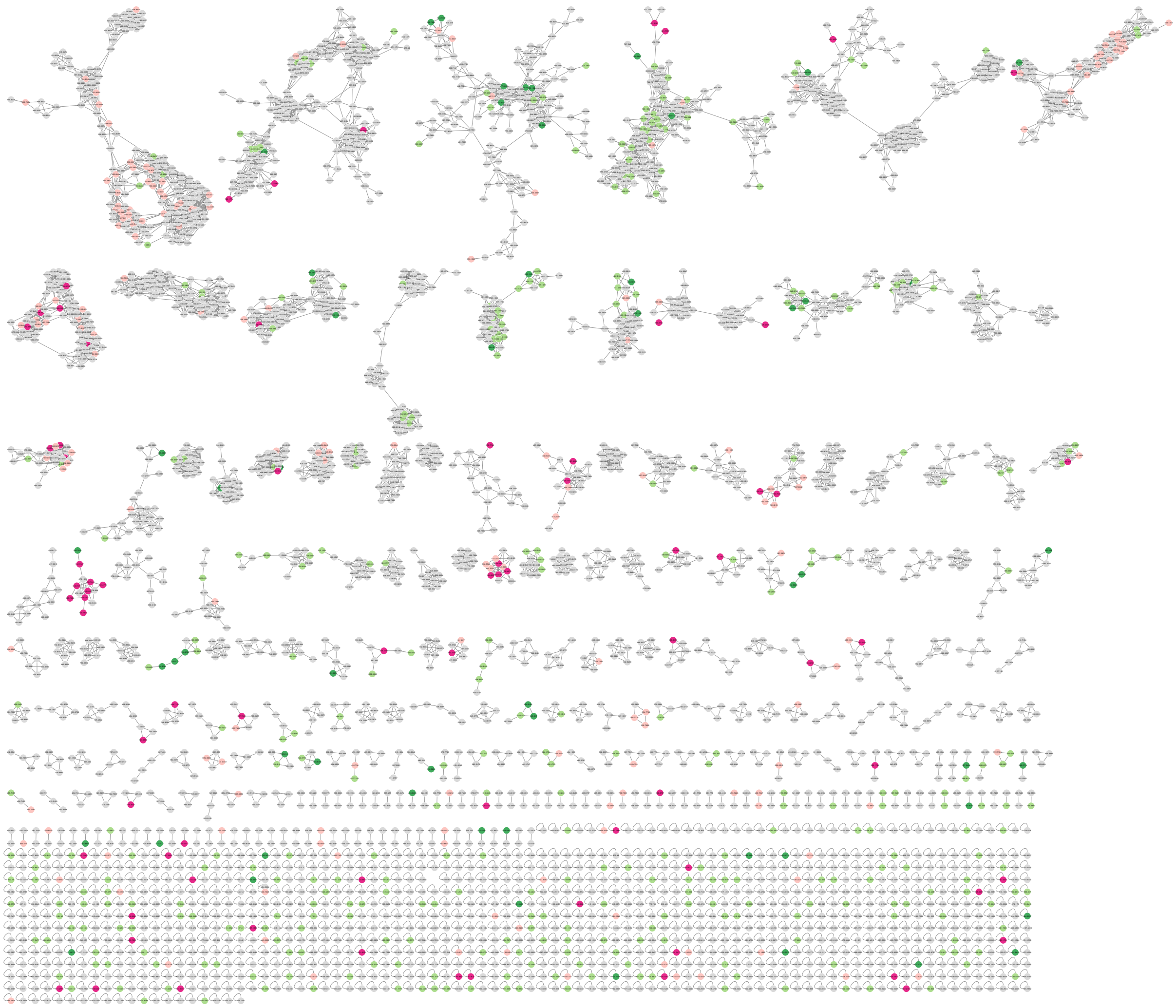

Figure S3. A) Positive and B) Negative mode molecular networks from GNPS. Correlation to pDA (pink) or Chl (green) at $p < 0.05$ (light) and $p < 0.01$ (dark). Nodes are labeled with precursor mass.

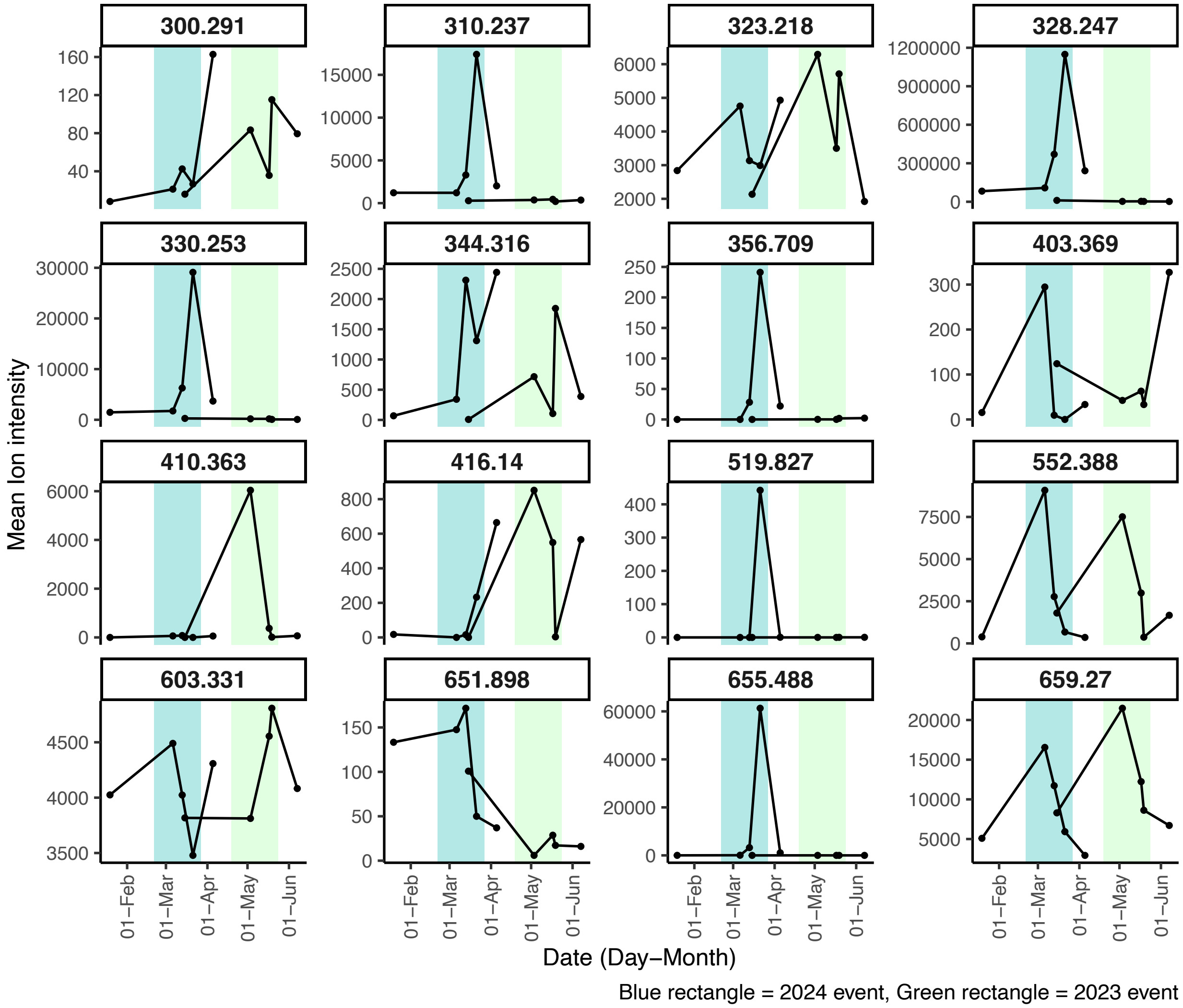

Figure S4. Cabrillostatin cluster mean ion intensity over time.

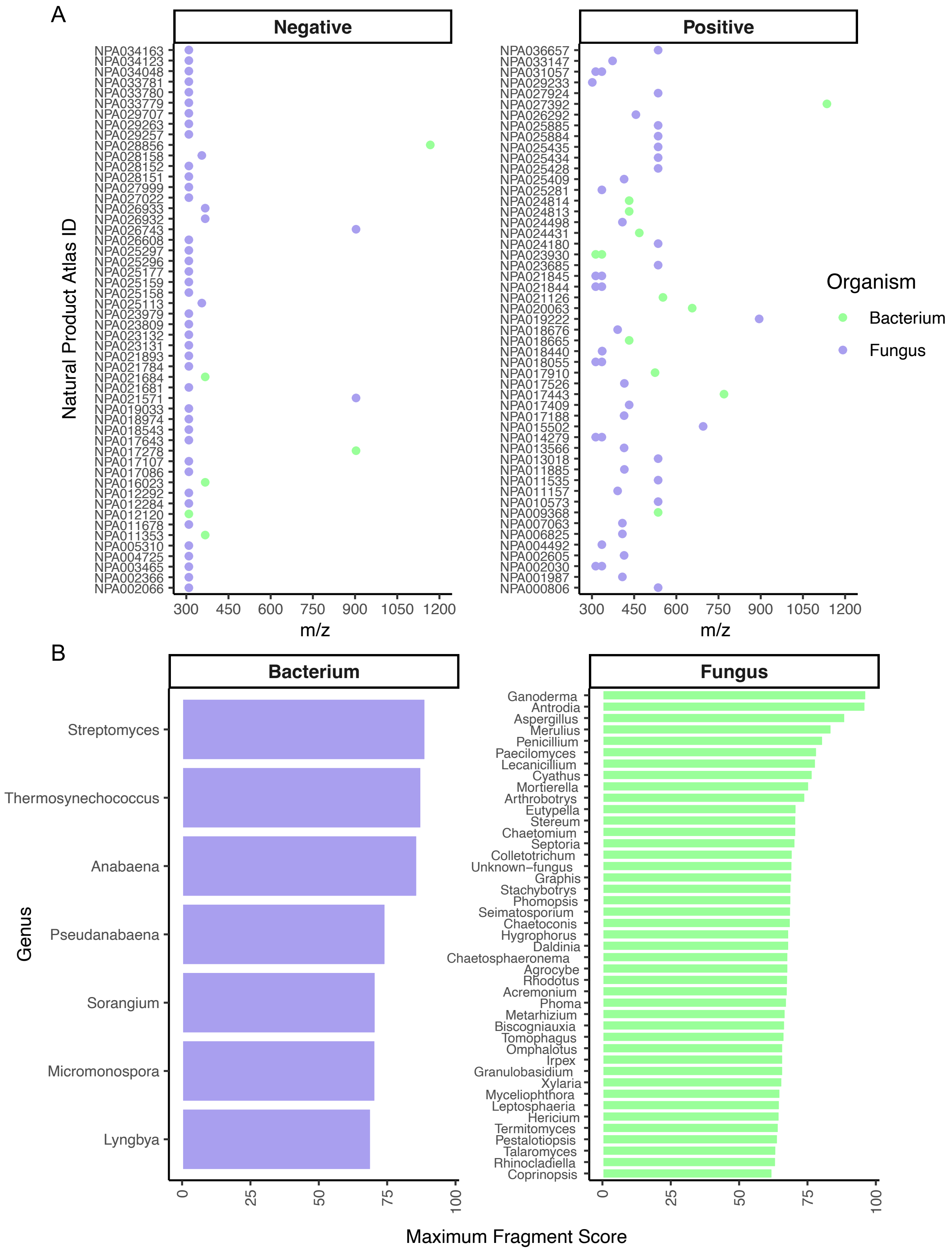

Figure S5. A) Potential identifications from Progenesis QI fragmentation algorithm and the Natural Product Atlas. B) Highest scoring genera from Bacteria and Fungi.

A

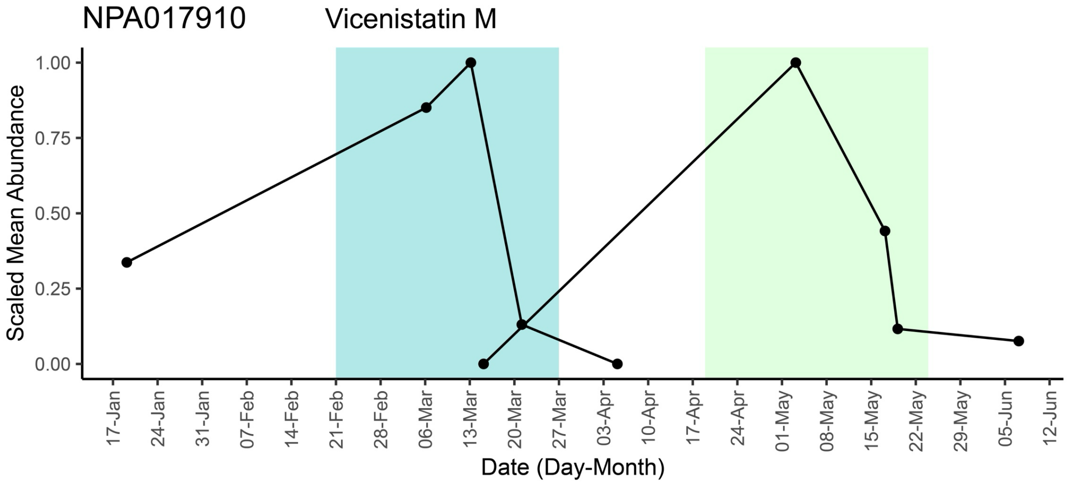

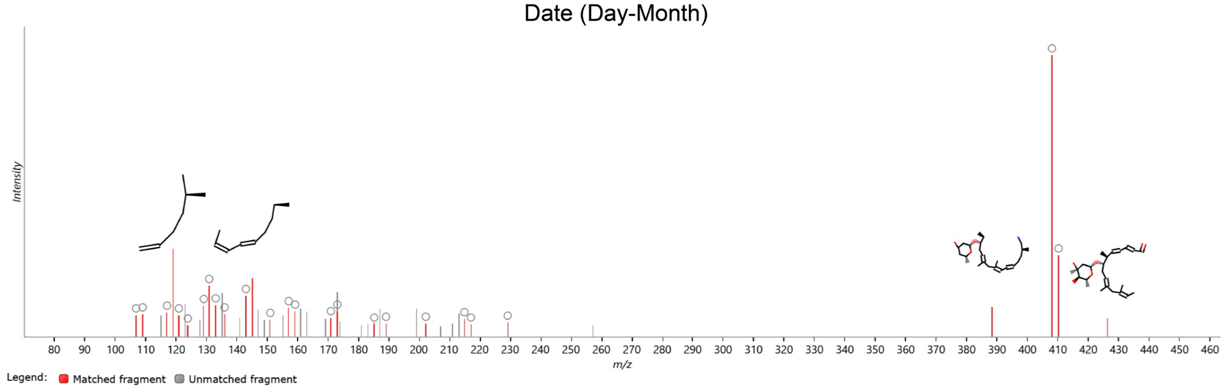

m/z	524.3338
Mass error (ppm)	-1.699
Retention time	3.66
Formula	C30H47NO5
Adducts	M+Na
Score/Frag Score	53.6/82.7
Isotope similarity	87.26
Origin	Bacteria
Genus	*Streptomyces*

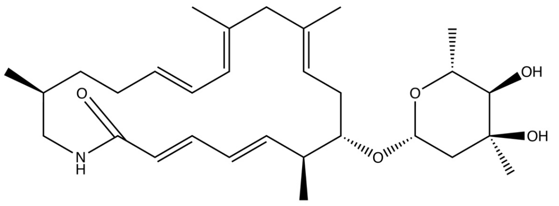

SMILES: C[C@H]1CC/C=C/C=C(/C/C(=C/C[C@@H]([C@H](/C=C/C=C/C(=O)NC1)C)O[C@H]2C[C@]([C@@H]([C@H](O2)C)O)(C)O)/C)\C

B

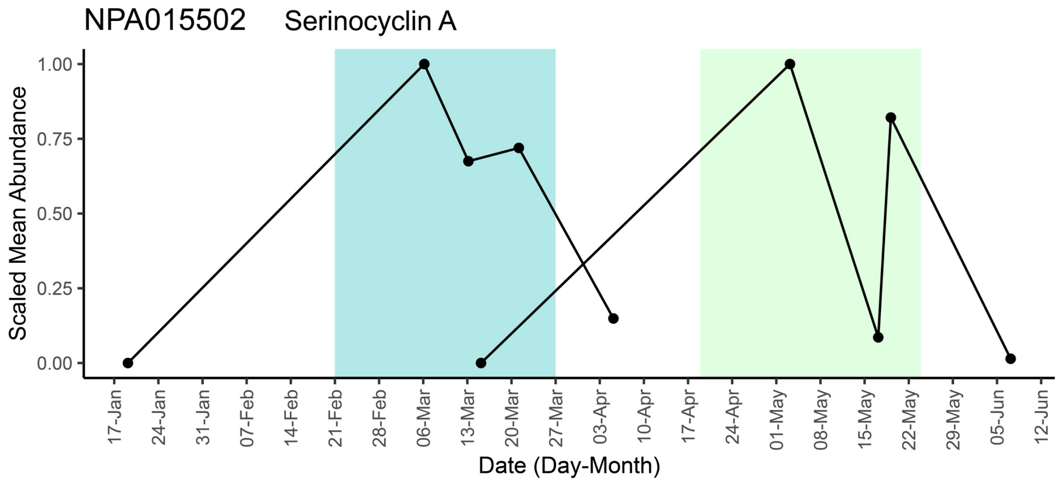

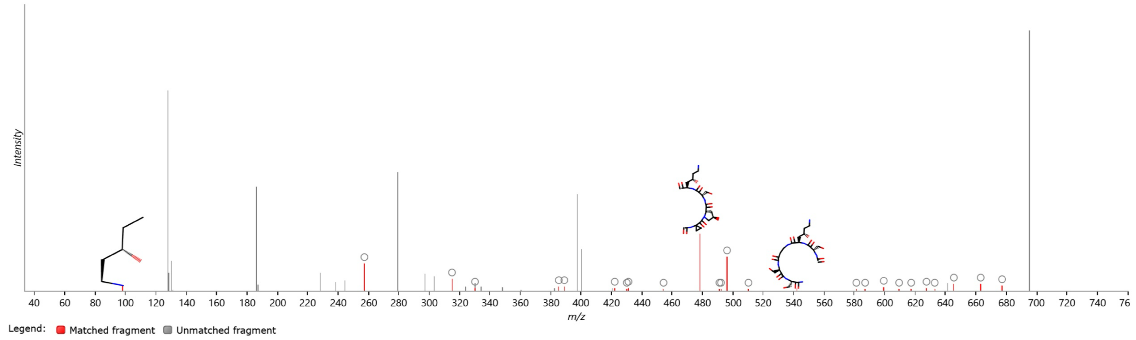

m/z	695.293
Mass error (ppm)	-5.184
Retention time	3.97
Formula	C27H44N8O12
Adducts	M+Na
Score/Frag Score	1.3/67
Isotope similarity	95.63
Origin	Fungi
Genus	*Metarhizium*

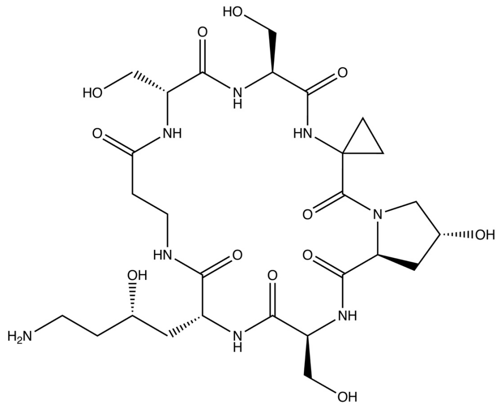

SMILES: C1CNC(=O)[C@H](NC(=O)[C@@H](NC(=O)[C@@H]2C[C@H](CN2C(=O)C3(CC3)NC(=O)[C@@H](NC(=O)[C@H](NC1=O)CO)CO)O)CO)C[C@H](CCN)O

Figure S6. Two features A) *m/z* 524.33 and B) *m/z* 695.29 with positive correlations to pDA were identified by Progenesis QI via theoretical fragmentation and the Natural Products Atlas. Yearly scaled SPATT detection, Progenesis QI fragment match, summary information, and chemical structure for potential match with bacterial compound, Vicenistatin M. Blue rectangle = 2024 event, green rectangle = 2023 event.

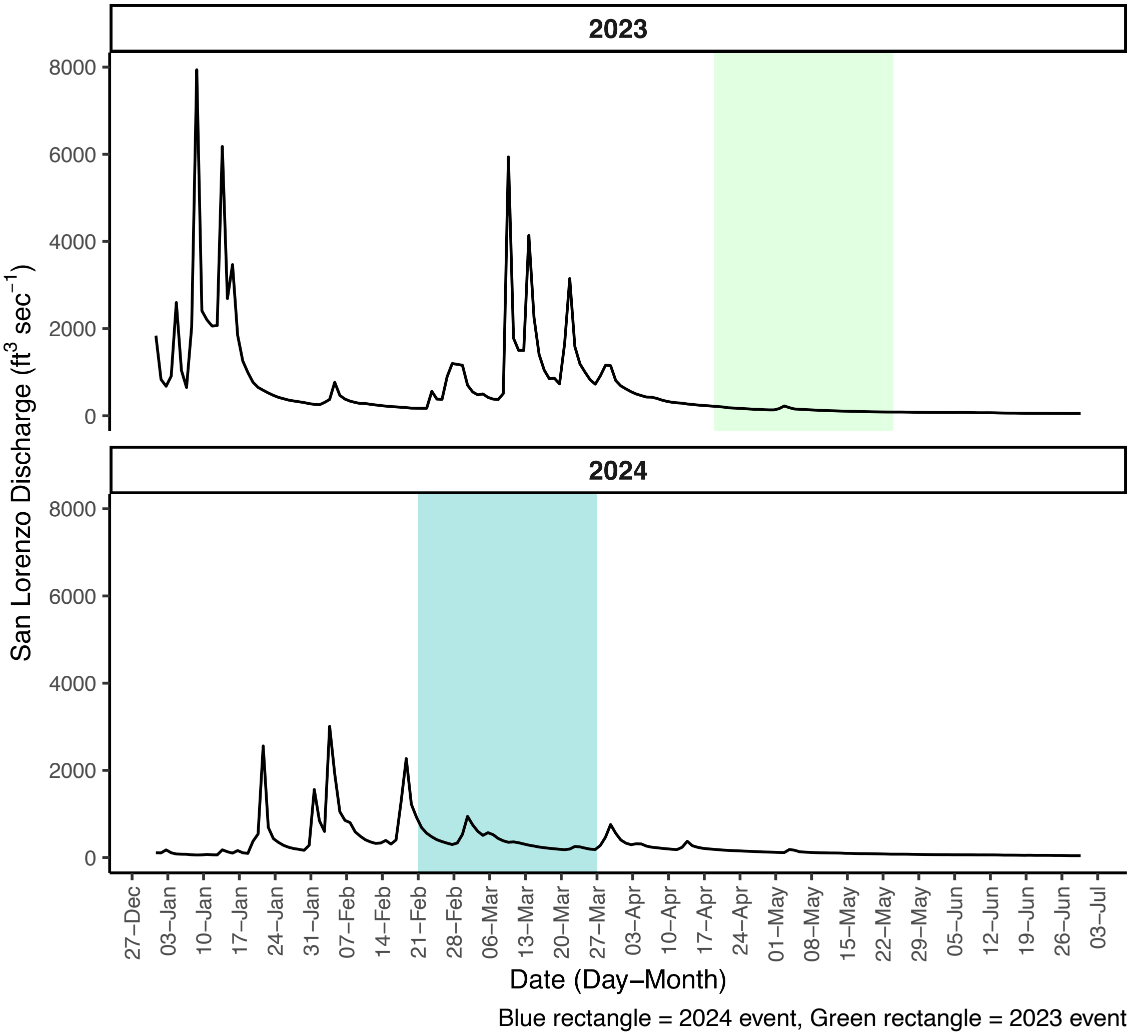

Figure S7. Flood gauge data from NOAA and USGS were used to observe freshwater discharge to the Santa Cruz Wharf from the San Lorenzo River.

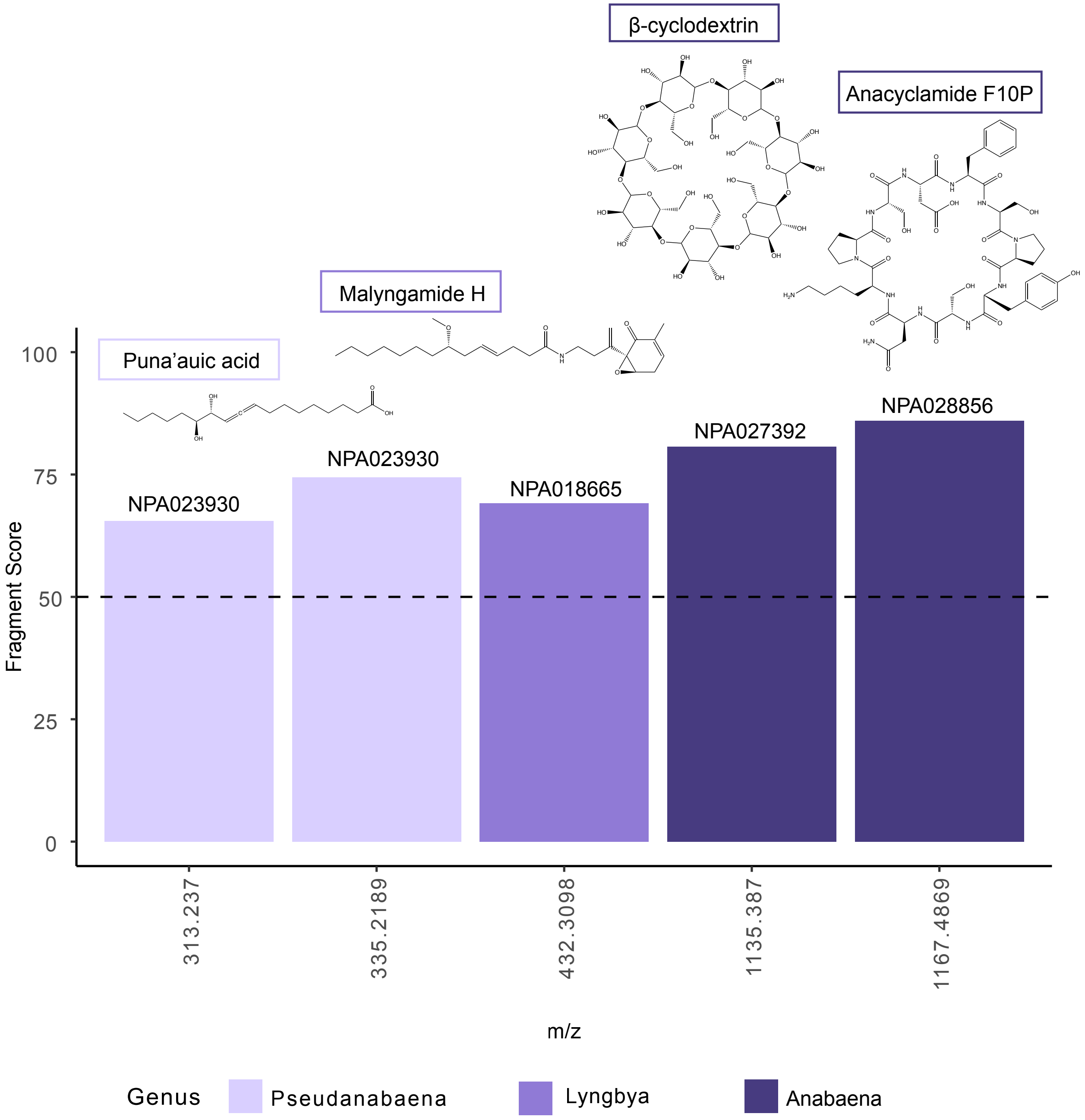

Figure S8. Potential matches from SPATT extracts to natural products from cyanobacteria.
